# Less Isn't Simple: Insights From Postmastectomy Radiotherapy Deintensification Trials

**DOI:** 10.1200/JCO-25-02852

**Published:** 2026-03-31

**Authors:** Robert W. Mutter, Karla V. Ballman

**Affiliations:** ^1^ Mayo Clinic School of Graduate Medical Education, Rochester, MN

There has been a longstanding interest in identifying lymph node–positive patients with breast cancer who can safely omit postmastectomy radiotherapy (PMRT). The recently reported results of the NSABP B-51/RTOG 1304 (B-51)^1^ and BIG 2-04 MRC SUPREMO (SUPREMO)^2^ trials were eagerly anticipated as potential milestones in addressing this unmet need and have been hailed by some as practice-changing. Here, we examine the clinical and statistical considerations underlying these trials as a framework for evaluating efforts to deintensify radiotherapy. Specifically, we explore whether B-51 and SUPREMO definitively rule out a benefit from PMRT in eligible patients, or whether they should be viewed as starting points for more nuanced, patient-centered decision making.

## PMRT Practice at the Outset of B-51 and Supremo

In the Early Breast Cancer Trialists' Collaborative Group (EBCTCG) meta-analysis, among 1,314 women with one to three positive nodes treated with adjuvant systemic therapy, PMRT significantly reduced overall recurrence (rate ratio [RR], 0.68 [95% CI, 0.57 to 0.82]) and breast cancer mortality (RR, 0.80 [95% CI, 0.67 to 0.95]).^3^ Importantly, PMRT in the seminal randomized trials included chest wall irradiation (CWI) combined with regional nodal irradiation (RNI), including axillary, supraclavicular, and internal mammary nodal basins.^4-6^

Advances in multidisciplinary care, including marked escalation of systemic therapy, have since improved outcomes for patients treated with upfront mastectomy. In parallel, neoadjuvant chemotherapy (NAC) has become routine for selected disease subtypes, and patients presenting with clinically node-positive disease who convert to pathologic node-negative status after NAC experience substantially lower recurrence rates than those with residual nodal disease, even without PMRT.^7^ These favorable outcomes led some to suggest that the absolute benefit of PMRT would be small and unlikely to translate into a survival advantage.^8^ Conversely, others argued that an excellent response to NAC may heighten the importance of durable locoregional control.^9^ These competing perspectives underscored the need for randomized trials to rigorously evaluate the safety of PMRT omission in selected low-risk patient populations treated with modern systemic therapy. Until such data emerged, prospective evidence supported National Comprehensive Cancer Network guideline recommendations to base PMRT decisions on the highest clinical or pathologic stage and to strongly consider PMRT, including both CWI and RNI, for node-positive disease.

## B-51 Design

**FIG 1.**
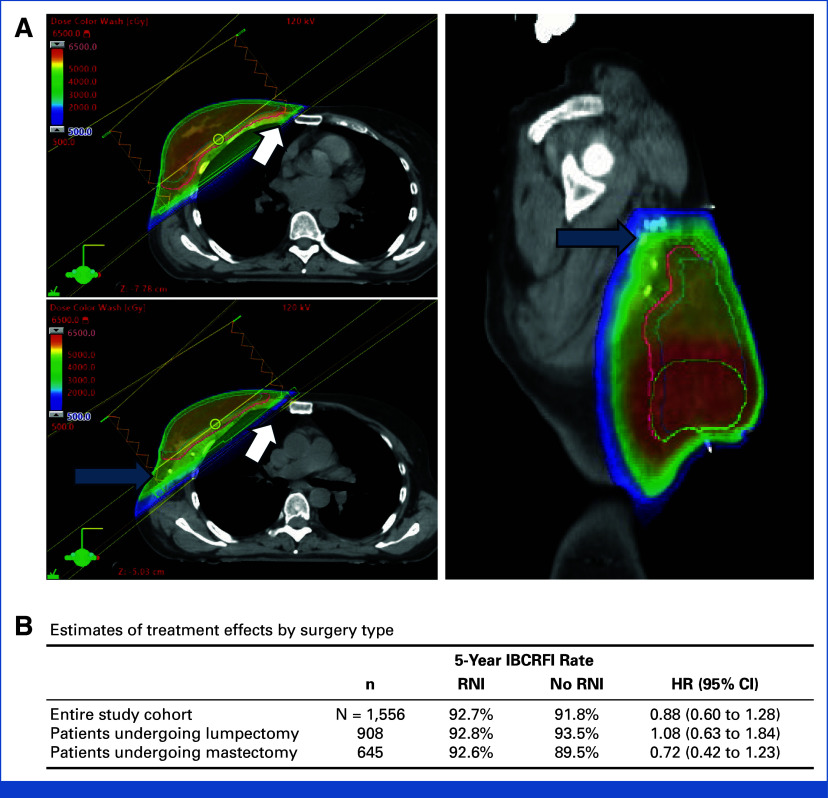
(A) Radiotherapy treatment plan for a patient treated with lumpectomy and randomly assigned to no RNI on the B-51 clinical trial. The axial (left) and sagittal (right) dose color wash images demonstrate clinically significant incidental exposure of the axillary (blue arrows) and internal mammary (white arrows) lymph node basins. Breast CTV (blue); breast PTV (pink); lumpectomy cavity PTV (green, right). (B) Estimates of treatment effects by surgery type in B-51. CTV, clinical target volume; HR, hazard ratio; IBCRFI, invasive breast cancer recurrence–free interval; PTV, planning target volume; RNI, regional nodal irradiation.

B-51 was designed to test the superiority of RNI compared with no RNI among patients presenting with cT1-3N1 breast cancer who achieved ypN0 status after NAC.^1^ Patients underwent lumpectomy or mastectomy with sentinel lymph node biopsy (SLNB) or axillary lymph node dissection (ALND).^1^ The primary end point was invasive breast cancer recurrence–free interval (IBCRFI). In the RNI arm, patients received RNI plus CWI after mastectomy, historically termed PMRT,^3-5^ or RNI plus whole-breast irradiation (WBI) after lumpectomy, resulting in comprehensive and consistent radiotherapy coverage across surgery approaches. Patients randomly assigned to no RNI received no radiotherapy after mastectomy, whereas those undergoing lumpectomy received WBI. Notably, WBI exposes substantial portions of axillary and even internal mammary nodes due to their proximity to the generous whole-breast target volumes mandated in the trial (Fig 1A).^10^ Thus, B-51 addressed two distinct clinical questions—one for lumpectomy and one for mastectomy—within a single study.

## SUPREMO Design

SUPREMO evaluated whether CWI following mastectomy and axillary staging improved 10-year overall survival (OS) compared with no CWI in a population treated almost exclusively with adjuvant systemic therapy.^2^ Eligible patients included those with pT1-2N1M0 and pT3N0M0 disease; patients with pT2N0M0 tumors were eligible if the tumor was grade 3 or exhibited lymphovascular invasion. All node-positive patients underwent axillary clearance. Importantly, RNI was not required, and fewer than 1.5% of patients in the CWI arm received comprehensive RNI, which now represents the standard of care for PMRT in node-positive disease.^11,12^

## A Priori Statistical Considerations

Both SUPREMO and B-51 were designed to test the *superiority* of radiotherapy over omission, rather than to establish *noninferiority* (NI) of omitting PMRT (ie, CWI + RNI), which was arguably the standard of care. Superiority designs aim to detect clinically meaningful benefit justifying treatment intensification, whereas NI designs are typically used when evaluating deintensification, with prespecified margins intended to prevent meaningful loss of benefit. NI margins are usually smaller than superiority margins because they are meant to prevent meaningful loss of benefit in a deadly disease; they fundamentally attempt to show that outcomes between the two treatments are similar within a small range of differences. Superiority margins are often larger because the intent is to demonstrate a clinically important, practice-changing improvement that justifies the burden (eg, adverse events, costs, inconvenience) of intensifying treatment. Trial size depends on the event rate in the control arm, the assumed effect size, and the chosen margin; although the sample size of an NI trial is often larger than that of a superiority trial, when event rates are low (eg, 5-year IBCRFI >90%), NI trials may require similar or even smaller sample sizes than superiority trials.

A meta-analysis previously found no benefit for PMRT in node-negative disease.^3^ Consequently, SUPREMO's inclusion of node-negative patients would be expected to increase the risk of falsely concluding that CWI does not meaningfully improve outcomes. Similarly, in B-51, combining mastectomy and lumpectomy cohorts in a single trial increases the risk of falsely concluding that PMRT does not reduce invasive recurrence, particularly as the proportion of lumpectomy patients increases (Figs 1A and 1B).^3,12^

## Subset Analyses

Both trials observed substantially fewer events than anticipated in their original designs. SUPREMO assumed 10-year OS rates of 56% without CWI and 63% with CWI. However, with 9.6 years of median follow-up, the observed 10-year OS was 81.8% in the no-CWI arm and 82.6% in the CWI arm reflecting the favorable risk profile of the population, which included 24.1% with pT2N0 and 40.0% with pT1-2N1 disease and a single micro- or macrometastasic node.^2^ B-51 planned its final analysis after 172 events but allowed early closure if fewer than 172 events occurred 10 years after trial initiation. Investigators elected to analyze outcomes after only 102 events, with a median follow-up of just 5.0 years. Among the entire B-51 study population, the 5-year IBCRFI was 91.8% in the no-RNI arm versus 92.6% in the RNI arm (hazard ratio [HR], 0.88 [95% CI, 0.60 to 1.29]; *P* = .51).^1^

There are established clinical differences between node-negative and node-positive patients (SUPREMO), and between lumpectomy and mastectomy cohorts (B-51). Accordingly, subset analyses can help guide clinical application of study findings, even if not powered for subgroup comparisons. In B-51, 648 (42%) of 1,556 patients underwent mastectomy. Although no evidence of benefit from RNI was observed among lumpectomy patients—potentially due to incidental irradiation of nodal regions at highest risk for microscopic disease (Fig 1A)^10^—a 28% relative improvement in 5-year IBCRFI was seen among mastectomy patients receiving PMRT (RNI): 92.6% versus 89.5% (HR, 0.72 [95% CI, 0.42 to 1.23]; Fig 1B).^1^ This effect did not reach statistical significance, but the trial was not powered for this subset analysis. Importantly, the observed HR aligns with the approximately 30% relative recurrence reduction reported in the EBCTCG meta-analysis, which concluded that “the proportional reductions in the rates of overall recurrence and breast cancer mortality (with PMRT) did not differ significantly according to whether or not systemic therapy was given or according to any other known tumor-related or treatment-related factor.”^3^ Importantly, because B-51 was a superiority trial, lack of statistical significance does not imply that omission of PMRT is noninferior to PMRT. Demonstrating NI would require a deintensification design with a prespecified NI margin—typically smaller than the 4.6% absolute difference assumed in B-51's design. Notably, the observed 3.1% absolute improvement in 5-year IBCRFI with PMRT exceeds NI margins commonly used in early-stage breast cancer trials (usually 2%-3%).^13,14^ Moreover, over half of the B-51 participants had hormone receptor–positive disease, a subgroup expected to maintain a similar or even higher annual recurrence risk 15 years or more beyond the initial 5-year follow-up.^15^ Among all (lumpectomy + mastectomy) patients with hormone receptor–positive disease, there was a numerical benefit of RNI (94.9% *v* 92.1%) that did not reach statistical significance.^1^

In SUPREMO, there was no clinical benefit among patients with node-negative disease treated with CWI, consistent with prior studies.^3^ However, among node-positive patients, CWI significantly reduced chest wall recurrence (HR, 0.30 [95% CI, 0.11 to 0.82]) and locoregional recurrence (HR, 0.51 [95% CI, 0.27 to 0.96]).^2^ Distant metastasis-free survival (DMFS) and disease-free survival (DFS) did not significantly differ between arms, including among node-positive patients. However, distant recurrence events by nodal status were not reported. With DMFS and DFS end points, any death is treated as a cancer event. Thus, in a population with a relatively low event rate and high rates of non–cancer-related mortality, small but clinically relevant differences in recurrence events may be obscured.

## Implications on Clinical Practice

The SUPREMO trial reaffirms that CWI is not indicated in patients with T1-T2 node-negative breast cancer treated with upfront surgery.^3^ These patients should not be included in future trials evaluating the safety of PMRT de-escalation.

For node-positive patients treated with upfront mastectomy, counseling remains more nuanced. Although data support a small but significant reduction in breast cancer mortality with PMRT in patients with low nodal burden, select patients with otherwise favorable clinical features may reasonably elect observation following mastectomy plus ALND to avoid potential adverse effects of PMRT.^11,12^ For many patients, however, PMRT may provide a better therapeutic ratio by improving local, regional, and distant disease control and potentially enabling omission of ALND.^12,16^

Because PMRT benefit in node-positive disease is largely driven by RNI,^12^ and CWI alone is now rarely used,^11^ the absence of mandated RNI in SUPREMO limits applicability to contemporary practice. The crude locoregional recurrence rate of 4.8% in node-positive patients in the no CWI arm likely underestimates the true cumulative incidence over time, and CWI reduced the risk of chest wall recurrence by more than two thirds.^2^ Nonetheless, the relatively small absolute benefit raises the hypothesis that RNI alone, without CWI, may be reasonable for select patients, particularly those with a single positive lymph node who comprised the majority of node-positive patients enrolled. Although total mastectomy was required in SUPREMO, this approach warrants further investigation in patients treated with skin- and nipple-sparing mastectomies with implant-based reconstruction, in whom the risks associated with CWI are greater. Of note, approximately 80% of the SUPREMO population had hormone receptor–positive disease. Investigators are encouraged to report detailed cumulative locoregional and distant recurrence events with longer follow-up, as well as subset analyses of invasive recurrence (eg, 1 *v* 2-3 positive nodes) among node-positive patients, to better inform shared decision making.

For those treated with NAC as in B-51, the absence of a benefit signal among the large lumpectomy cohort supports protocol-defined WBI (Fig 1A) without RNI for most eligible patients. For the smaller mastectomy cohort, B-51 data indicate no statistically significant improvement in IBCRFI with PMRT. Some patients may reasonably elect observation.^11^ However, B-51 does not provide strong evidence of a lack of benefit of PMRT in eligible patients given the superiority trial design, enrichment of lumpectomies, relatively short follow-up, and approximately 30% relative reduction in recurrence rate observed.^1^ Personalized decision making should incorporate consideration of pathologic response in the breast and axillary management. Indeed, only 22% of B-51 participants failed to achieve a breast pathologic complete response, and in this subgroup, RNI was associated with a nonsignificant 26% reduction in recurrence. Likelihood of adherence to recommended adjuvant systemic therapy may be an especially important consideration in these patients. Axillary management further complicates interpretation. ALND was performed in 45% of patients; however, axillary management by surgery type was not reported. With stronger evidence for reducing axillary surgery in patients with positive sentinel nodes receiving breast-conserving therapy (BCT),^10,16^ most mastectomy patients likely had ALND. Notably, RNI was associated with a 25% lower recurrence rate among all (lumpectomy + mastectomy) patients treated with SLNB.^1^ Although this difference did not reach statistical significance, the clinical implications of a false-negative SLNB may be particularly consequential for patients undergoing mastectomy who do not receive adjuvant radiotherapy (Fig 1A). Moreover, the cN1 eligibility criterion encompasses a wide spectrum of nodal disease, ranging from a single to multiple clinically involved axillary nodes. Detailed reporting of baseline clinical characteristics of mastectomy patients, as well as recurrence outcomes among mastectomy patients treated with SLNB, would provide important additional context for interpreting the B-51 trial results.

We owe a debt of gratitude to the patients who participated in B-51 and SUPREMO. These trials set the stage for future studies incorporating novel predictive biomarkers to tailor locoregional therapy.^17,18^ When deintensification of the standard of care is pursued, NI designs should be used, with margins informed by both clinician judgment and patient perspectives.^19^ Notably, many patients treated with mastectomy in B-51 and SUPREMO may have been suitable candidates for BCT. In B-51, BCT was associated with the highest IBCRFI regardless of RNI (Fig 1B). This, along with robust data supporting the omission of ALND in BCT patients with positive sentinel nodes,^10,16^ reinforces BCT as the preferred approach to optimize the therapeutic ratio for most suitable patients.^20^ Ultimately, shared decision making grounded in each patient's values regarding recurrence risk reduction relative to potential treatment-related morbidity should form the basis for identifying the optimal personalized treatment strategy.
